# The Effects of Age, Dosage, and Poverty on Second Language Learning through SparkLing^TM^ in Infant Education Centers in Madrid, Spain

**DOI:** 10.3390/ijerph182312758

**Published:** 2021-12-03

**Authors:** Naja Ferjan Ramírez, Kaveri K. Sheth, Patricia K. Kuhl

**Affiliations:** 1 Department of Linguistics, University of Washington, Seattle, WA 98195, USA; ksheth2@uw.edu; 2 Institute for Learning & Brain Sciences, University of Washington, Seattle, WA 98195, USA; pkkuhl@uw.edu; 3 Department of Speech and Hearing Sciences, University of Washington, Seattle, WA 98195, USA

**Keywords:** L2, bilingual, infancy, intervention, LENA, early education centers

## Abstract

The first 1000 days represent a unique window of opportunity for second language learning. In two recent studies we demonstrated that Spanish infants’ use of second-language (L2) English productive vocabulary and early utterances rapidly increased through the play-based, interactive and highly social SparkLing^TM^ Intervention, which consists of an evidence-based method and curriculum stemming from decades of research on infant language development. Analyzing an expanded and more diverse sample of Spanish infants (*n* = 414; age 9–33 months) who received the SparkLing^TM^ Intervention, this study examines the variability in L2 production, which was assessed via first-person LENA audio recordings. Infants’ age significantly and positively correlated with L2 production, demonstrating an advantage for older infants in the sample. While overall socioeconomic status (SES) was not related to L2 production, very young infants (under two years) who lived in high poverty homes showed faster increases in English production compared to peers who lived in moderate poverty homes. Infants’ attendance in the program (“dosage”) was also predictive of their L2 production outcomes. Infants across SES have the capacity to begin acquiring two languages in early education classrooms with SparkLing^TM^ through one-hour/day sessions in social environments that engages them through frequent high-quality language input.

## 1. Introduction

Bilingualism brings about many advantages, such as communicating with a greater number of people, expanding one’s social circles, increasing travel and work opportunities, appreciating of other cultures, experiencing greater access to services, and increasing earnings [1,2,3,4]. Studies have also identified specific cognitive advantages [5], although this work has been challenged [6] and more follow-up research is needed (see also [7]). There is now abundant evidence that bilingualism brings about a number of positive social advantages, such as peer cooperation, and the ability to understand information in multiple ways [8,9]. Perhaps unsurprisingly, learning and using an additional language has been shown to have an impact on the structure and function of the brain, including in regions involved in language, cognitive control, and the connections between them [10,11,12].

### 1.1. School-Based Programs for L2 Instruction

Many communities worldwide have been creating school-based programs for early second language (L2) acquisition. In recent years, factors such as age, length and intensity of exposure, didactic approach, and teacher proficiency have been associated with children’s early L2 school-based learning outcomes [13,14,15,16]. The effectiveness of these programs has been highly variable. However, when interpreting the findings of these studies, it is important to keep in mind that the vast majority of studies consider children who already have a well-established first language (L1) and receive L2 instruction through a single non-native speaker in a fairly limited context (see [17,18]). For various societal, educational, political, and economic reasons, L2 access is still limited for the vast majority of infants and toddlers (i.e., children under the age of 3 years). L2 learning in infant education classrooms is limited in many parts of the world, including the United States, Latin America, Asia, and Europe, even though many communities have adopted bilingual education curricula at the grade school level, and occasionally in preschools (i.e., for children aged 3–5 years; [18,19,20]). Infant bilingual education tends to only be offered through small private programs, which are often out of reach to most families. This is unfortunate, given that the infant brain is uniquely prepared to learn multiple languages [21,22]. Behavioral studies in the laboratory indicate that infants have a well-documented ability to rapidly, naturally, and efficiently acquire one or multiple languages through social interactions (see [23] for review). 

In 1996, Spain made a commitment to bilingual (Spanish-English) education through immersion in public schools by signing an agreement with The British Council [24]. In the Madrid metro area, the commitment to bilingual education was particularly strong, and the goal was to eventually adopt bilingual education in 30% of high-schools, 50% of elementary schools, and 100% of preschools (serving children 3–6 years of age; [25]). In 2014, a pilot program for bilingual immersion education was also introduced in a small number of Madrid’s public infant education centers (escuelas infantiles), serving children aged between years. However, while the grade-school bilingual program in Spain was based on guidelines and evaluation practices from The Council of Europe [26]; few, if any, guidelines were available for establishing an infant bilingual program, and the evaluation of its success was limited to observation. 

In the academic year 2015/2016, we launched a collaboration with Madrid’s Ministry of Education, Youth, and Sports, with the goal of evaluating their infant L2 program, and testing an alternative, scalable, research-based approach through the SparkLing^TM^ Intervention, described in detail in [27] (henceforth “2017 Study”) and [28] (henceforth, “2020 Study”). In the section below, we briefly summarize the findings of these two studies. 

#### Second Language Learning in Madrid’s Public Infant Education Centers

The goal of our two SparkLing^TM^ Intervention studies in Madrid was to assess how much and what kind of foreign language exposure is necessary to ignite L2 learning in the context of infant education centers. In both cases, we tested a six-point language learning method combined with a weekly theme-based language curriculum, both of which reflected a theoretical model and a long history of research documenting the principles of infant language development ([23,29]; outlined below). SparkLing^TM^ Intervention children were compared to carefully matched Control children in the same schools who experienced Madrid’s existing Infant Bilingual Program, which focused on activities that were already part of the school’s existing everyday (Spanish) curriculum (henceforth referred to as Current Practice Comparison, CPC), with the English portion consisting of approximately 2 h of weekly instruction led by a Spanish-English trained bilingual teacher, who introduced simple English vocabulary and phrases through activities such as book reading, nursery rhymes, and singing. All of the English teachers in the CPC program held the Cambridge B1 or B2 Certificate; however, their English was highly accented. The infant schools had English books, and other English materials, such as posters and wall decor with English words, which were utilized by the teachers during the English instruction period (see [27] for details). 

In both studies, we assigned children (*N* = 250 in the 2017 Study, *N* = 240 in the 2020 Study; age range: 7–33.5 months) to the Intervention and CPC groups, matching age, sex, and baseline Spanish and English levels. Four different public infant education centers (schools) participated in each study; they were located in different neighborhoods of the Madrid metro area, and served local families, which varied in their socio-economic status (SES). In both studies, children in the Intervention and CPC group attended the same schools. Intervention children participated in daily, 1-h (2017 Study) or 45-min (2020 Study) English play sessions (SparkLing^TM^ Intervention), led by teams of language “tutors,” who were native English speakers and undergraduate students or recent graduates of the University of Washington. Prior to the start of the Intervention, all of the tutors participated in an in-person (2017 Study) or online (2020 Study) training, during which they received instructions about the research principles behind the Intervention, as well as instructions on how to utilize the SparkLing^TM^ curriculum to execute the Intervention through daily group play sessions. 

The 6 principles of the language Intervention, and the research behind them, are described in detail in [27,28]. In brief: (1) Tutors provided a high quantity of English input. (2) During the Intervention sessions, tutors used “parentese,” which is characterized as having higher pitch, slower tempo, and exaggerated intonation contours. (3) The learning context was highly social with weekly themes, games, and activities that prompted face-to-face interaction. (4) Tutors were trained to provide prompt, contingent responses and to socially engage the children in frequent back and forth exchanges. Children were encouraged to “talk” and interact. (5) Children heard English from multiple native speakers (3–4 tutors worked with 12–20 children at a time). (6) The daily sessions were presented through adult-scaffolded play and language content was delivered in a way that ensured distributed exposure.

While both studies implemented the same 6-point language Intervention, there were some critical differences between their implementation: most notably, in the 2020 Study, the tutor training was delivered through an interactive online training and certification tool, the purpose of which was to provide the tutor training in scalable, accessible, and transferrable way. Other differences included a lower tutor/child ratio, shorter daily sessions, lower overall SES in the 2020 Study (see also Table 1 in [28] for more details).

In both studies, the initial Spanish levels were assessed by the European Spanish MacArthur-Bates Communicative Developmental Inventory (CDI; [30]). Spanish and English comprehension was assessed with the Computerized Comprehension Task (CCT; [31]). English production outcomes were assessed using the LENA (Language ENvironment Analysis) technology, in combination with manual annotation of children’s English utterances. In both studies, several steps were taken to ensure that the research procedures imposed as little interference as possible with infants’ day-to-day needs, activities, and the schools’ curricula. For example, rather than dividing individual children into the Intervention and the CPC group by random assignment, as would be required by a classical Randomized Control Trial design, all of the participating children in a given classroom were assigned to the same group, either Intervention or CPC, which did not differ on any demographic or language characteristics at the start of the study [27,28].

Results demonstrated that the Intervention children in both studies showed rapid gains on measures of English comprehension and production, significantly outperforming the CPC group on all L2 measures. While both the SparkLing^TM^ Intervention and the CPC groups showed some growth in English word comprehension over 18 weeks, the Intervention group outperformed the CPC group by a large margin (*p* < 0.001; Cohen’s *d* = 1.34 in 2017 Study, and 0.74 in the 2020 study). For language production, the difference between the Intervention and the CPC groups was even more pronounced: At the end of the 18-week period, the SparkLing^TM^ Intervention group produced an average of 74 English vocalizations per child per hour in the 2017 Study and 49.4 English vocalizations per child per hour in the 2020 Study, while CPC children in Week 18 averaged at 13 (2017 Study) and 14.4 (2020 Study) English vocalizations per child per hour, respectively, (Intervention Group Cohen’s *d* = 1.79 or a 6-fold increase from Week 1 to Week 18 in the 2017 Study, and *d* = 1.19 or a 4.7-fold increase from Week 1 to Week 18 in the 2020 Study). While the growth in English comprehension and production was significant in both studies, it was somewhat attenuated in the 2020 Study compared to the 2017 Study. As we explain in greater detail in [28], this was likely, in part, due to shorter daily English lessons in the 2020 Study compared to 60 min per day in the 2017 study. 

Finally, it is important to note that, in both studies, children’s L1 (Spanish) comprehension grew equally in the Intervention and in the CPC groups, showing that Spanish language learning was *not* affected by participation in the SparkLing^TM^ Intervention. In follow-up testing, we investigated the degree to which Intervention children retained their English knowledge after the conclusion of the Intervention through an additional English comprehension assessment, conducted 18 weeks after having returned to their usual (CPC) classrooms. Children’s English comprehension scores did not decline (or grow) during this period, demonstrating that the effects of English learning were retained at least 18 weeks after the completion of the Intervention. 

### 1.2. The Present Study

Our previous results demonstrate that infants are capable of making rapid gains in L2 learning in the context of an early education setting. However, while children’s L2 growth in the 2017 and in the 2020 Study was rapid *on average*, we observed a fair amount of variability, especially in children’s English production. In the present study, we take a closer look at some of the factors potentially contributing to this variability. Focusing on children’s English *production*, the present study has two aims: 

Aim 1: The 2020 Study reported results from four infant education centers, in order to match the demographic characteristics of the 2017 Study sample and draw comparisons between the tutor training delivered in person (2017 Study) vs. online (i.e., through the online training program, 2020 Study). However, in the 2020 Study, English language data over the first 18 Weeks of the study (September–February) was collected at 7 schools (*N* = 414). The first aim of the current study is to present the combined results on children’s English production between Week 1 and Week 18 across all 7 schools, seeking to replicate the 2020 Study results on a larger and more diverse sample of participants, which allows us to consider the potential effects of children’s age, SES, and program attendance (“dosage”) on children’s English production. Our hypotheses are based on our previous findings, and are outlined below:

- Age: In the 2020 Study, we divided our sample into younger children (under 18 months) and older children (over 18 months) and reported that, on our main measure of English production, older learners outperformed younger learners. This finding was expected, given that we are studying children’s English production. Within the age range studied (9–33 months), older children are generally more talkative than younger children. We anticipate replicating this finding in the current study across a larger sample, which allows us to study the effects of age as a continuous variable (i.e., entered in days), hypothesizing that it will correlate positively with children’s English production;
- Neighborhood Wealth (henceforth, socioeconomic status, SES): In the 2017 and 2020 studies, we reported that the school’s neighborhood wealth (SES) had no impact on children’s English language production. In both published studies, the four participating schools varied in SES from mid to low. In the present study, we consider 7 schools, which constitute a broader SES range, and includes one school where the rate of poverty in children’s homes was extremely high. According to Madrid’s Ministry of Education, Youth, and Sports and the school’s principal, most children attending this school lived in conditions of extreme poverty and were part of a community of migrant workers in which parental literacy and education skills were very low.
  As in our previous studies, we use the percentage of children receiving free meals at the schools as an SES-proxy. Based on our previous findings, we hypothesize that SES will not have an impact on children’s English language production, even across this broader SES range;
- Dosage (“Class Attendance”) Effects: In the 2017 Study, Intervention children received one hour of English exposure per day. This was reduced to 45 min per day in the 2020 Study, and we observed that English production was somewhat attenuated. We proposed that shorter English language sessions may have been one reason for this attenuation. In the present study, we consider the potential effects of dosage defined as children’s actual English session attendance scores, which reflect the amount of English language input that children received. These data, which were gathered each day by the language tutors, varied substantially in the sample. We hypothesize that children who attended more English sessions will show higher English language production scores.

**Figure 1:**
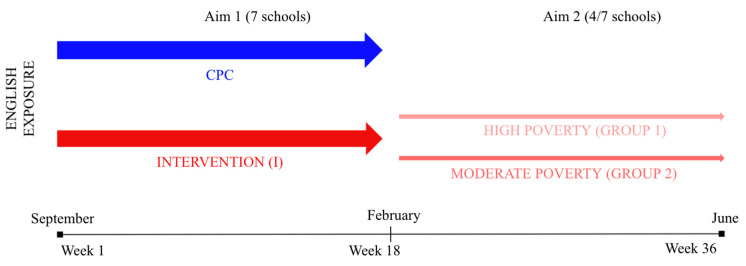
Outline of study design, I, SparkLing^TM^ Intervention; CPC, current practice comparison.

Aim 2: The second aim of the current study is to conduct a follow-up analysis of children’s English production in 4 schools where the Intervention continued beyond the first half of the school year, spanning the entire school year (36 weeks; Figure 1). We specifically focus on the potential effects of extreme poverty in the home (very low SES) on children’s English language production at school over the full 36 weeks by comparing one school were poverty was very high (migrant worker community, low parental education and literacy rates, all children meet eligibility criteria to receive free meals at the school), to three other schools where poverty was moderate. While there is a wealth of literature documenting the negative effects of poverty on children’s L1 development, little is known about how severe poverty in the home may impact children’s L2 learning in a school context. Previous classroom research with older children indicates that there are significant correlations between students’ SES and their academic achievement [32], including L2 learning [33]. For example, [34] found a strong positive relation between parents’ educational levels (one factor contributing to SES measures) and primary school students’ foreign language learning in Hungary. Along similar lines, [35] reported that Chinese students’ English performance differed by their parents’ educational levels. However, in both cases, the children were older than in the present sample, and some of the proposed mechanisms that may have contributed to the observed variability were attributed to factors that may not apply to infants (such as self-efficacy, self-perceived competence, motivation). On the other hand, school-based interventions aimed to enhance children’s language learning in infancy and toddlerhood often report *larger* effect sizes for children who begin the programs with the lowest language levels [36], who often come from low SES backgrounds. There is also evidence that children who enter an intervention program earlier perform better at language assessments [36,37] supporting the idea that language interventions may be particularly effective for youngest learners who represent the lowest SES backgrounds. With these seemingly conflicting previous findings in mind, we did not have any specific hypotheses as to the rate of L2 learning in the extremely high vs. moderate poverty-level schools; however, we noted that infants’ age may play an important role when considering their rates of benefiting from the Intervention. 

## 2. Materials and Methods

### 2.1. Participants and Schools

Parents of 438 infants aged between 9 and 33 months at the start of the study signed informed consent. Enrollment criteria included no major birth or postnatal complications, and no significant exposure to English from live human speakers outside of school. Data from 24 children were excluded after review of the background questionnaires, due to parental report of suspected or confirmed developmental delay (*n* = 9) or English exposure through live speakers outside of the school (*n* = 15). A total of 414 children had usable data for analyses. The ethics committee at the University of Washington approved the study. 

The seven participating schools served families who lived in their neighborhoods, which ranged in SES from mid to extremely low. In the Community of Madrid, infant education is optional, and private and public centers are available. Admission to public centers (the schools in the present study) is based on a points system, taking into consideration the families’ income and presence of any adverse social circumstance, with the goal of admitting local families most in need of affordable childcare. Children from families whose yearly per capita income is below €4260 are eligible to receive free meals at the school. In the present study, the percentages of children from such families were: SchoolA: 11%, SchoolB: 35%, SchoolC: 48%, SchoolD: 64%, SchoolE: 76%, SchoolF: 79%, SchoolG: 100%. It is important to note that in SchoolG, the rates of poverty were extremely high. According to the Madrid Ministry of Education, Youth, and Sports, this school differed from the rest of the participating schools in that children not only met the criteria to receive free meals at the school, but far exceeded them. Furthermore, according to the school’s principal, most families in this school came from a community of migrant workers where literacy and education levels were very low. As in our previous work [28], the free-meal percentages were used as an SES-proxy in our analyses of the effects of poverty. While the relationship between SES and language development is well documented, there is currently no consensus on the most effective measure of SES (for review, see [38]). As the SES index was collected at the level of the school (rather than individual families), and because it was entirely income-based, it is not an ideal tool as it may not capture the additional hardships experienced by the families who live in extreme poverty (such as SchoolG). However, it has the advantage of avoiding the collection of potentially very sensitive information, especially in families of very low SES backgrounds where literacy and education levels may be very low. 

### 2.2. English Exposure

All Intervention children experienced daily, 45-min long English play sessions led by teams of 3 tutors, who interacted with a group of 14 children (in classrooms for ages 9–21 months) or 20 children (in classrooms for ages 21–33 months). Tutors were undergraduate students or recent graduates and had successfully completed the online SparkLing^TM^ program. The two eligibility requirements were being a current or past undergraduate student at the University of Washington and being a native speaker of English (self-report, confirmed with an in-person interview). The interactive online program consisted of tutor training, testing, and eventual certification. Once trained, tutors received a set of weekly theme-based curriculum lesson plans, and a collection of classroom materials used to execute the curriculum lesson plans. The tutor training consists of five units describing the six-point method (described above, see also [27,28]) through interactive activities and video examples of tutor behaviors and children’s responses. Quiz questions appear throughout the training to provide knowledge checks. Each unit ends with a certification test that must be passed to progress to the next unit. Tutors received access to the 18-week or 36-week curriculum (lesson plans), describing how to implement the research principles through 45-min daily sessions, frequently referencing the research principles. The SparkLing^TM^ Intervention materials are a collection of children’s books and classroom manipulatives to support the classroom activities. A detailed description of the six key principles of the Intervention method, and the research behind each, are described in [27,28]. 

### 2.3. Language Measures 

The same methods were used as described in [28]. In brief, prior to Week 1, children’s Spanish level was assessed with the European Spanish MacArthur-Bates Communicative Developmental Inventory (CDI; [30]). Prior to Week 1, children who were at least 16 months of age participated in a Spanish and English vocabulary comprehension assessment with the Computerized Comprehension Task (CCT; [31]), an assessment of word comprehension administered on a touch screen. We have previously reported that Intervention and CPC children’s levels of Spanish were matched at the start and at the end of the Intervention, and this was replicated here (all *p*s > 0.1). We do not use these measures in the present analyses, but the baseline measures can be found in Supplemental Materials.

The main measure used in the present study is children’s rate of produced English vocalizations, assessed by manual annotation of audio recordings collected with the LENA technology (LENA Pro Version 3.4.0, [39]). Each child wore a LENA vest, with a digital language processor (DLP): a small, light-weight recorder placed into the vest’s pocket, designed to record the child’s voice and the language they hear. Children’s language production contained English and Spanish, in addition to other sounds, such as babble, raspberries, and imitations of animal sounds. To assess children’s English production, manual “coding” (annotation) was performed, and all statistical analyses were conducted on manually coded data. The fully analyzed dataset reported here includes the LENA recordings from Week 1 (September) and Week 18 (February) for Intervention children across 7 schools, Week 18 (February) recordings for CPC children, and Week 36 (June) recordings for a subset of Intervention children in 4 out of 7 schools who continued to receive the Intervention between Week 18 (February) and Week 36 (June). To reduce the interference with the school’s curricula, the CPC group was not assessed with LENA in Week 1.

Details about LENA data collection, coding, and analysis procedures are described in [27,28]. Briefly, coders identified all of the English vocalizations in a 25-min segment of each child’s recorded file. As in the 2017 and 2020 Study, statistical analyses were performed on the total number of English vocalizations per child per hour, obtained by multiplying the number of English vocalizations in the 25-min segment by 2.4. All LENA data was logarithmically transformed to meet the assumptions for parametric testing, determined by the skewness and kurtosis values, both of which were between −2 and +2, which is considered acceptable in order to prove normal univariate distribution [40,41,42].

## 3. Results

The results section is divided into Aim 1 (replication of 2020 Study results with a larger sample, effects of age, SES, and attendance), and Aim 2 (effects of poverty in the home on children’s English language production). 

### 3.1. Aim 1

**Figure 2:**
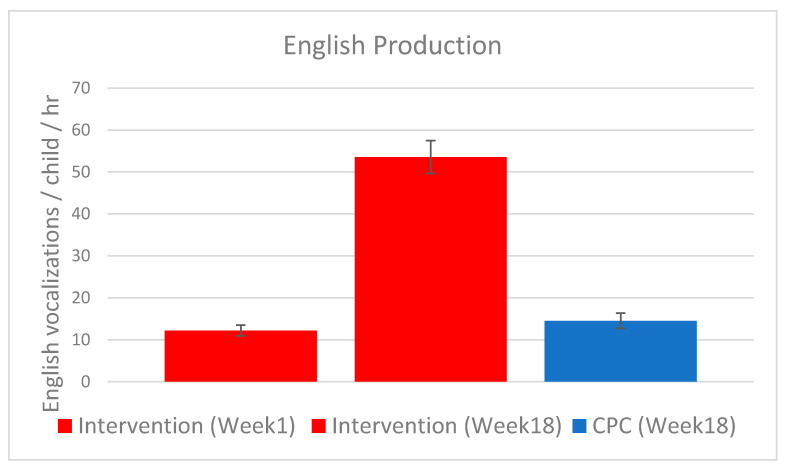
Mean number of English vocalizations per child per hour for Intervention group (red; *n* = 267) at the Week 1 and Week 18 measurement points, and for the CPC group (blue; *n* = 108) at the Week 18 measurement point. Error bars represent standard error.

A total of 267 Intervention children had valid LENA recordings in both Week 1 and Week 18. In order to compare the rate of English vocalizations from the same participant at different timepoints (Week 1 and Week 18), a paired t-test was used. As shown in Figure 2, the rate of English vocalizations per child per hour changed significantly from Week 1 (*M* = 12.2, *SD* = 20.72) to Week 18 (*M* = 53.6, *SD* = 63.50), *t*(266) = 15.02, *p* < 0.001, *d* = 1.04, 95% CI [0.57, 0.74]. In Week 18, 108 CPC children had a valid LENA recording. An independent t-test was used to compare the number of English vocalizations from the Intervention group and the CPC group. An independent t-test was chosen in order to compare the means in two different groups at the same timepoint. In Week 18, the Intervention group produced more English vocalizations per child per hour than the CPC group (*M* = 14.5, *SD* = 18.9), *t*(373) = 6.77, *p* < 0.0001, *d* = 0.77, 95% CI [0.37, 0.68]. These results replicate our findings from the 2020 Study, indicating that the Intervention children’s increase in English production over the 18-week period was substantial. 

#### Effects of Age, SES, and Attendance on SparkLing^TM^ Intervention Children’s English Production

A total of 279 Intervention children had a valid LENA recording in Week 18. Of these, 2 children attended fewer than 10 English sessions, and were excluded from the follow-up analyses. Our first step in these follow-up analyses was to consider the potential effects of age, entered as a continuous variable (i.e., age in days at the start of the study). As hypothesized, using a Pearson correlation since both variables were continuous and quantitative, age was significantly and weakly positively correlated with children’s number of produced English vocalizations at the Week 18 measurement point, *r*(277) = 0.49, *p* < 0.001. 

Controlling for age, we then asked whether SES, entered as the percentage of students at each school that receive free meals, correlated with children’s number of produced English vocalizations at the Week 18 measurement point. As hypothesized, using a Pearson correlation since both variables were continuous and quantitative, SES was not significantly correlated with children’s number of produced English vocalizations in Week 18, *p* > 0.1. 

We next considered the effects of dosage (“class attendance”), which ranged widely in the present sample, from having attended a total of 19 English sessions over 18 weeks, to a total of 69 English sessions over 18 weeks (*M* = 57.50 sessions; *SD* = 9.1 sessions). Controlling for age, we considered the relation between cumulative attendance (i.e., number of total sessions attended over 18 weeks) and children’s number of English vocalizations produced at the Week 18 measurement point. 

Using a Pearson correlation since both variables were continuous and quantitative, attendance was significantly and weakly positively correlated with children’s number of English vocalizations in Week 18, *r*(276) = 0.19, *p* = 0.001. Since cumulative attendance and children’s number of English vocalizations in Week 18 correlated, in order to examine whether attendance predicted children’s number of English vocalizations at the Week 18 measurement point, a 2-stage hierarchical multiple regression was conducted. This model was chosen in order to be able to control for age while using regression. A child’s number of produced English vocalizations at Week 18 was the dependent variable. Age was entered at stage one of the regression to control for the age in days at the start of the study. Cumulative attendance up to Week 18 was entered at stage 2. The hierarchical multiple regression revealed that at stage one, age contributed significantly to the regression model, *F*(1, 277) = 89.86, *p* < 0.001, and accounted for 24.5% of the variation in children’s number of English vocalizations at the Week 18 point. Introducing the cumulative attendance up to Week 18 explained an additional 2.8% of variation in children’s number of English vocalizations at the Week 18 measurement point and this change in R^2^ was significant, *F*(1, 276) = 10.59, *p* = 0.001, demonstrating that cumulative attendance is a unique incremental predictor of variation in a child’s number of English vocalizations after 18 weeks in the SparkLing^TM^ Intervention program. When both independent variables were included in stage two of the regression model, both age and attendance were significant predictors of a child’s number of English vocalizations at the Week 18 measurement point. 

### 3.2. Aim 2

At the Week 18 measurement point, the Intervention group was split into 2 subgroups (Figure 1): children in 4 of the participating schools (Schools B, D, E, and G) continued receiving the SparkLing^TM^ Intervention for another 18 weeks, until the end of the school year, while the children in the remaining three schools returned to their usual classrooms and continued with the CPC program. Of those who stayed in the Intervention, 45 children attended the school where the rate of poverty in children’s homes was extremely high (SchoolG, 100% of children on free meals; migrant worker community with low literacy and education rates), and 145 children attended the other three schools where the rate of poverty in children’s homes was moderate (SchoolB 35% on free meals, SchoolD 64% on free meals, SchoolE, 76% on free meals). In the analyses below, we consider children’s English production at the Week 1 measurement point, the Week 18 measurement point, and the Week 36 measurement point, by comparing children from SchoolG (Group1) to those from SchoolsB, D, and E (Group2). Children in Group1 and Group2 were matched in age (*p* = 0.49). 

We considered all children in Group1 (*n* = 45) and Group2 (*n* = 145) who had a valid LENA recording at each of the 3 timepoints. Independent t-tests at each timepoint were used to compare the average number of English vocalizations produced per child per hour of Group1 and Group2. There were no significant differences between the two groups at any one of the timepoints, Week 1: *t*(181) = 1.60, *p* = 0.111; Week 18: *t*(166) = 1.16, *p* = 0.249; Week 36: *t*(175) = 1.58, *p* = 0.119, although Group1 children were numerically above Group2 children at each timepoint. 

To consider the potential effects of children’s age in our analyses, we divided the participants into Younger (0–733 days old; *n* = 94) and Older (734–1004 days old; *n* = 96) children using a median split. A median split was used in these analyses due to the relatively low number of children in Group1 (School G; 45 children; 21 younger, 24 older). An independent t-test was conducted at each timepoint in order to compare the average number of English vocalizations per child per hour of the Older children in Group1 and Group2 who had a recording at each of the three timepoints. There were no significant differences between Group1 and Group2 in the average number of English vocalizations per child per hour at any one of the timepoints, Week 1: *t*(92) = 0.88, *p* = 0.379; Week 18: *t*(81) = 0.351, *p* = 0.726; Week 36: *t*(84) = −0.23, *p* = 0.82. An independent t-test was used to compare the means of the Younger children in Group1 and Group2 who had a valid LENA recording at each of the three timepoints. There were no significant differences between the two groups in the average number of English vocalizations per child per hour at Week 1, *t*(25.996) = 1.22, *p* = 0.235, or Week 18, *t*(83) = 0.33, *p* = 0.327. However, there was a significant difference between Group1 (*M* = 1.69, *SD* = 0.36) and Group2 (M = 1.43, SD = 0.65) at Week 36, *t*(57.634) = 2.33, *p* = 0.023, with Group1 children producing a significantly *higher* number of English vocalizations per child per hour compared to Group2 children. That is, after 36 weeks of SparkLing^TM^ Intervention, the younger children from high poverty homes produced a significantly higher number of English vocalizations per child per hour compared to age matched children from homes where poverty was moderate. 

## 4. Discussion

The present study had two aims. The first aim was to replicate the results from a previous study with a larger and more diverse sample of participants, focusing on factors potentially affecting the variability in their L2 production. The second aim was to take a closer look at the potential effects of poverty in children’s homes on their L2 production in a school context. The general pattern of results replicates our previous work (i.e., the 2017 Study and the 2020 Study). That is, the children who received the SparkLing^TM^ Intervention outperformed the CPC group by a large margin in terms of their English production. As hypothesized, older children in the sample produced a greater number of English vocalizations compared to younger children. Further, SES was not related to a child’s English production, even though the range of SES was much broader in the present study, and included one school where poverty was extremely high. Together, these results demonstrate that infants, across a wide range of SES backgrounds, are capable of making rapid gains in L2 learning in the context of an early education setting. However, they also underscore the fact that the nature and quality of foreign language programs for infants and toddlers play a critical role in learning. Infants can begin acquiring a second language rapidly, but the environment must socially engage them through high quality and quantity language input. 

The present study has important implications for the implementation of the SparkLing^TM^ program for school-based L2 instruction in infancy and beyond. With the measures examined here, older children in the study did not show a learning disadvantage, but instead showed faster learning compared to younger learners. In part, this may be because the focus of the present study is on higher levels of language (speech production at the word and utterance level); it is possible that interesting differences between younger and older learners do exist at the level of phonetic learning, where younger learners have been shown to outperform older learners [29,43]. However, within the age range studied here, older learners are more physically and cognitively mature compared to their younger counterparts and may be at an advantage for word and/or utterance-level learning, as has been previously demonstrated in internationally adopted children [44]. For example, it may be that older L2 learners may require fewer exposures to establish links between words and concepts; they may also be more likely to successfully encode the L2 input or retain it. Improvements in general cognitive skills, such as attention and memory, could also play an important role in accelerated word-learning that is typically observed in L1 during the second and third years of life; the present findings suggest that, if frequent high-quality input is available, fast, and efficient word learning during this period can be extended to L2 learning. 

The present findings also suggest that the SparkLing^TM^ Intervention could be adapted for use with older learners in the 3–5-year-old age-range. Of course, children in the preschool age range (3–5 years) require different language scaffolding activities than infants, suggesting that the present Intervention should be carefully modified to accommodate older learners. Furthermore, it is important to acknowledge that previous studies have shown age-dependent variability for acquisition of grammatical morphology within the preschool age (see [45]), suggesting that the learning constraints on older children may be distinct from what we observed in the present sample, and should first be scientifically investigated with interventions similar to the present one. Nevertheless, in many countries around the world (for example, the United States), early education centers cater to older children [46], suggesting that methods for classroom L2 instruction in the preschool age range may be more scalable than those intended for infants. 

Another important finding in the present study concerns the effects of dosage, defined here as children’s actual class attendance for the English sessions. Perhaps surprisingly, given the vast variation in how early L2 programs are implemented across the world [47], relatively little is known about how the amount of input affects children’s language learning; while this is true for older children, it is especially true for younger learners. Further, studies that have looked into this question in the past have typically considered only the number of offered hours of instruction, as opposed to the number of hours that children actually attend (for example, [19]). As demonstrated in the present dataset, children’s attendance, and therefore exposure to L2 input, can vary drastically, even in the same classroom. 

Of course, it is important to note that other related factors that we did not investigate here could also affect the variability in children’s rates of L2 acquisition. One good candidate to further investigate is the tutor to child ratio. Teacher-child ratios are often used as quality indicators in early childhood education and care [48], and some studies show that better ratios are associated with improved child outcomes, particularly language [49,50]. Furthermore, research indicates that exposure to multiple speakers enhances L1 learning [51,52]. While these findings suggest that a higher tutor to child ratio may lead to better L2 outcomes in the context of infant education centers, this question is yet to be tested experimentally. As the present intervention was based on six principles, we cannot yet specify the minimal conditions that are necessary for producing the observed effects. We also cannot predict how changing one of the parameters (for example, the tutor to child ratio) may affect the results. Follow-up studies focusing on a single variable at a time will be necessary to answer such questions. 

It is also important to acknowledge that due to the relatively short duration of the Intervention and the fact that participating children were very young, the current study focused on the early stages in infant language production and did not go beyond the assessment of volubility of infants’ English speech. Further research will be needed to determine whether research-based interventions in early education settings can in fact create truly bilingual minds. Within the present dataset, follow-up analyses of diversity of children’s English vocabularies are currently underway. New intervention studies with preschool aged children and/or spanning longer periods of time will be needed to further examine children’s use of diverse grammatical structures, to study growth in children’s sentence length and complexity, and to assess children’s performance on standardized language tests. 

The present data demonstrate, to our knowledge for the first time, that the amount of L2 input infants receive in early education settings directly affects their L2 production outcomes. The relation between the amount of language input and children’s language outcomes is well established for L1 learning: The seminal work by Hart & Risley [53] demonstrated that children whose parents talk less tend to have smaller vocabularies by the time they are three years old. Our current findings support the existence of a similar relation between language input and learning in L2, and underscore the importance of frequent, high quality social interactions for language learning. Although infants have the incredible ability to quickly learn one or multiple languages, the quantity and quality of language input that they receive plays a critical role in this process (see [54,55]). 

Finally, the present findings have important implications for infant L2 learners of varying SES backgrounds. Children from a range of SES backgrounds, including environments where poverty in the home is high, have the capacity to acquire one or two languages, provided that frequent high-quality language stimulation is provided through social interaction. Our previous studies have demonstrated that L2 learning through SparkLing^TM^ was effective regardless of children’s SES backgrounds. The present study replicates this finding with a broader SES range, including one school where poverty was extremely high and 100% of the children qualified for the free food program, in addition to facing a number of other hardships, according to the school’s principal and Madrid’s Ministry of Education, Youth, and Sports. Children in this particular school learned L2 at rates comparable to children attending other schools; that is, when considering infants across the entire age-range tested (9–33 months), there were no significant SES-related differences in their L2 production rate, although children from the high-poverty school were numerically above those from moderate-poverty schools. Interestingly, however, when children were split into two age groups, younger infants from the high-poverty homes showed *faster* L2 learning compared to their age-matched peers from schools where the levels of poverty were moderate. Similar findings have previously been documented for successful L1 interventions (see for example [36]), suggesting that intervening early can set low-income children on more positive trajectory for developmental success. The present study extends these findings to L2, suggesting that children raised in high-poverty homes have the capacity to successfully acquire two languages, provided that socially engaging high-quality language input can be provided in both languages in school. 

## 5. Conclusions

Many communities worldwide aspire to create successful programs for L2 instruction. By systematically investigating the variability inherent in programs to create the best early language learning classrooms, the present study represents an important step towards formulating well-founded recommendations for how best to implement early L2 instruction in schools. Children, including those from very low socio-economic backgrounds, have the capacity to begin second language learning in early childhood—they make rapid gains from social language exposure when the method of language exposure incorporates key evidence-based features that have been demonstrated to be effective in laboratory research [27]. Here we demonstrate that the quantity of L2 input plays an important role in learning, in addition to children’s age. Future studies should expand and adapt the current program for use in preschool environments, and systematically investigate its effects on children’s L1 and L2 learning via carefully designed intervention studies adapted for older children (aged 3–5 years). 

## Data Availability

Data is available at: https://osf.io/xr5fh/?view_only=1a6c9bd8130f44a9b30bdcfe8ae70564 (accessed on 21 September 2021).

## Supplementary Materials

The following are available online at https://www.mdpi.com/article/10.3390/ijerph182312758/s1, Supplemental Results.
