# Supplementary material for: The Effects of Age, Dosage, and Poverty on Second Language Learning through SparkLingTM in Infant Education Centers in Madrid, Spain
Source: Int J Environ Res Public Health. 2021 Dec 3;18(23):12758. doi: 10.3390/ijerph182312758 (PMC8656938; doi:10.3390/ijerph182312758)
Supplement: Supplementary file 1 [file ijerph-18-12758-s001.zip › ijerph-1410531-supplementary.pdf]

Supplemental Results

Baseline measures of Spanish

Prior to Week 1, children's Spanish level was assessed with the European Spanish MacArthur-Bates Communicative Developmental Inventory (CDI; [30]). A total of 380 families (275 Intervention, 105 CPC) returned valid CDI questionnaires. Baseline Spanish productive vocabularies ranged between 0 and 573 words, with the mean productive vocabulary of 140.4 (*SD* = 130.3) words. These scores placed children between 1st and 99th percentile for their age, with the average being the 31.5th percentile. The mean CDI productive vocabularies and percentile scores did not differ between the Intervention and the CPC group, $t(377) = 0.74$, $p = .46$, 95% CI [–22.6, 49.8] and $t(377) = -1.29$, $p = .20$, 95% CI [–10.22, 2.12]. These results replicate our findings from our previous studies [27, 28].

Week 1 and Week 18 measures of Spanish and English comprehension

Figure S1 shows mean English and Spanish CCT scores for Intervention and CPC children in Week 1 and in Week18. For English, 155 participants (118 Intervention, 37 CPC) had a valid score in both Week 1 and Week 18. The mean English CCT scores did not differ between the two groups at baseline ($t(153) = 0.178$, $p = 0.86$). A repeated measures ANOVA with Time (September/February) and Group (I/CPC) as independent variables showed a significant main effect of Time, $F(1, 153) = 65.35$, $p < .0001$ (Greenhouse-Geisser), partial $\eta^2 = .30$ and an interaction of Time and Group, $F(1, 153)= 6.25$, $p = .014$ (Greenhouse-Geisser), partial $\eta^2 = .04$, indicating the impact of the Intervention on the pattern of change over time. The interaction remained significant after controlling for SES (covariance analysis): $F(1, 152) = 5.62$, $p = .019$, partial $\eta^2 = .036$. For Spanish, 178 participants (138 Intervention, 40 CPC) had a valid score in both, September and February. There were no differences between the two Groups at baseline

($t(176) = 0.36$, $p = 0.71$). There was a main effect of Time, $F(1, 176) = 127.0$, $p < .0001$ (Greenhouse-Geisser), partial $\eta^2 = .42$, but the interaction of Time and Group was not significant, $F(1, 176)= 0.46$, $p = .70$ (Greenhouse-Geisser), partial $\eta^2 = .001$. The Spanish CCT scores in the two groups, as predicted, increased in both groups equally over the course of the 18-week Intervention period after starting at similar levels. These results replicate our findings from the 2020 Study.

Supplemental Figure S1: Mean English (top panel) and Spanish (bottom panel) Computerized Comprehension Task (CCT) Scores in Week 1 (September) and Week 18 (February), for the Intervention and CPC children who completed the assessments at both time points. Error bars represent standard error.

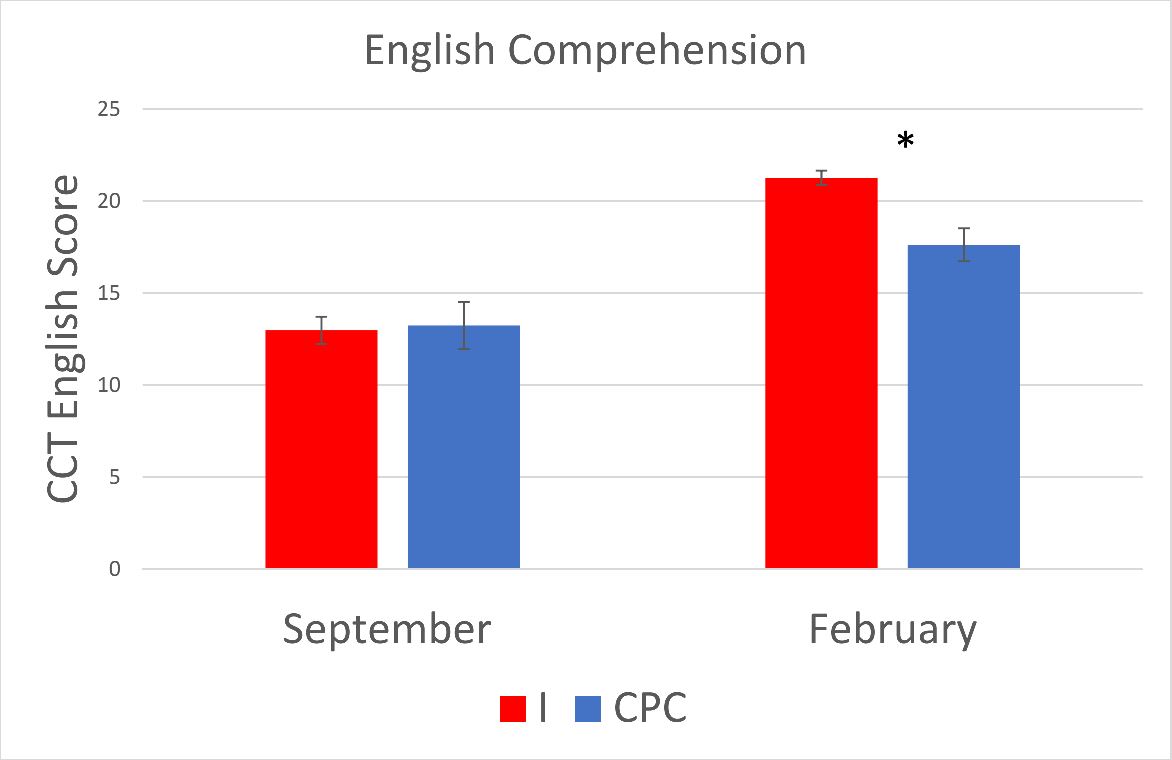

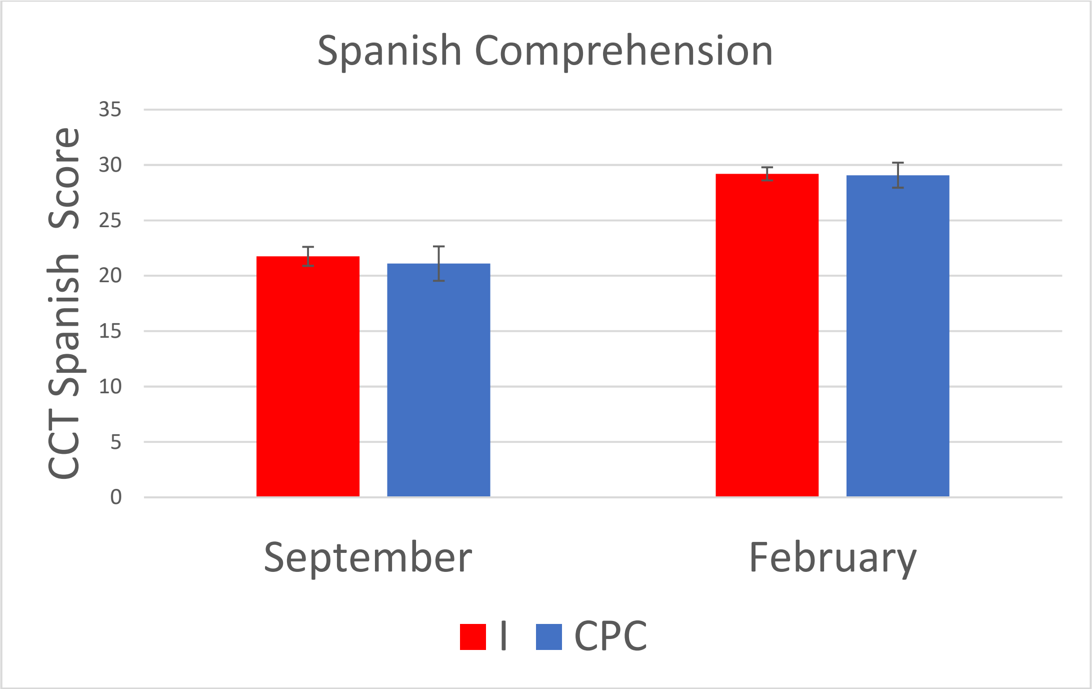
Spanish Comprehension
CCT Spanish Score
35
30
25
20
15
10
5
0
September
February
I
CPC
